# A mathematical model of neuroinflammation in severe clinical traumatic brain injury

**DOI:** 10.1186/s12974-018-1384-1

**Published:** 2018-12-18

**Authors:** Leah E. Vaughan, Prerna R. Ranganathan, Raj G. Kumar, Amy K. Wagner, Jonathan E. Rubin

**Affiliations:** 1 Department of Physical Medicine and Rehabilitation, University of Pittsburgh, 3471 Fifth Ave., Suite 202, Pittsburgh, PA 15213 USA; 2 Department of Mathematics, University of Pittsburgh, 301 Thackeray Hall, Pittsburgh, PA 15260 USA; 3 Safar Center for Resuscitation Research, University of Pittsburgh, Pittsburgh, PA USA; 4 Department of Neuroscience, University of Pittsburgh, Pittsburgh, PA USA; 5 Center for Neuroscience, University of Pittsburgh, Pittsburgh, PA USA

**Keywords:** Biomarker, Cerebrospinal fluid, Cytokines, Glasgow outcome scale, Inflammation, Mathematical modeling, Microglia, Patient outcome, Traumatic brain injury

## Abstract

**Background:**

Understanding the interdependencies among inflammatory mediators of tissue damage following traumatic brain injury (TBI) is essential in providing effective, patient-specific care. Activated microglia and elevated concentrations of inflammatory signaling molecules reflect the complex cascades associated with acute neuroinflammation and are predictive of recovery after TBI. However, clinical TBI studies to date have not focused on modeling the dynamic temporal patterns of simultaneously evolving inflammatory mediators, which has potential in guiding the design of future immunomodulation intervention studies.

**Methods:**

We derived a mathematical model consisting of ordinary differential equations (ODE) to represent interactions between pro- and anti-inflammatory cytokines, M1- and M2-like microglia, and central nervous system (CNS) tissue damage. We incorporated variables for several cytokines, interleukin (IL)-1β, IL-4, IL-10, and IL-12, known to have roles in microglial activation and phenotype differentiation. The model was fit to cerebrospinal fluid (CSF) cytokine data, collected during the first 5 days post-injury in *n* = 89 adults with severe TBI. Ensembles of model fits were produced for three patient subgroups: (1) a favorable outcome group (GOS = 4,5) and (2) an unfavorable outcome group (GOS = 1,2,3) both with lower pro-inflammatory load, and (3) an unfavorable outcome group (GOS = 1,2,3) with higher pro-inflammatory load. Differences in parameter distributions between subgroups were ranked using Bhattacharyya metrics to identify mechanistic differences underlying the neuroinflammatory patterns of patient groups with different TBI outcomes.

**Results:**

Optimal model fits to data showed different microglial and damage responses by patient subgroup. Upon comparison of model parameter distributions, unfavorable outcome groups were characterized by either a prolonged, pathophysiological or a transient, sub-physiological course of neuroinflammation.

**Conclusion:**

By developing a mathematical characterization of inflammatory processes informed by clinical data, we have created a system for exploring links between acute neuroinflammatory components and patient outcome in severe TBI.

**Electronic supplementary material:**

The online version of this article (10.1186/s12974-018-1384-1) contains supplementary material, which is available to authorized users.

## Background

The debilitating impact of traumatic brain injury (TBI) affects the lives of an estimated 10 million athletes, soldiers, and civilians every year around the world [1]. Despite shared physical, social, and economic burdens, these individuals experience unique patterns of brain damage and physiological responses following injury [2, 3]. Potent anti-inflammatory treatments have generally been unsuccessful for such a diverse patient population [4–6]. This issue of heterogeneity renders TBI treatment an immense clinical challenge due to known variability in the (1) patient population, (2) initial injury severity, (3) secondary injury mechanisms, and (4) emergent co-pathologies [3, 6, 7].

Neuroinflammation has long been identified as a secondary injury mechanism following TBI and is emerging as a major contributor to chronic neurological pathologies and outcome [2, 5]. Recent work has investigated the multifaceted role of neuroinflammation post-TBI [3, 6, 8, 9] in attempts to elucidate heterogeneity in the response that drives different patient outcomes. Resident central nervous system (CNS) microglia, which are immediately activated after traumatic insult and propagate the innate neuroinflammatory response, are implicated with injury-induced neuroinflammation as multi-dimensional responders. Research guided by previous macrophage studies suggests that microglia are selectively polarized via cytokine signaling to a spectrum of functional phenotypes ranging from a classic pro-inflammatory M1-like state to an anti-inflammatory M2-like state. Each phenotypic state has its respective role in the neuroinflammatory sequence including phagocytosis of damaged and dysfunctional neurons, neurogenesis, tissue repair and restoration, and immune regulation [2, 5, 10]. Distinguishing these functional phenotypes in humans by in vivo imaging techniques (such as positron emission tomography) is still in its infancy [11]. Alternative methodologies are warranted to complement these technological advancements and to characterize the temporal progression of microglial activation and functionality post-injury.

Characterizing the temporal dynamics of interdependent inflammatory cytokine cascades following injury is a critical step toward understanding the interplay between physiological neuroprotection and pathological neurotoxicity with respect to acute neuroinflammation and microglia activity [9, 12]. Mechanistic mathematical modeling can be an effective framework for this goal because it provides a representation of the simultaneous evolution of inflammatory mediators. Ordinary differential equation (ODE) models, in particular, have been commonly used to investigate the time-dependent interactions in complex inflammatory networks [13–17]. Equations are derived to represent specific biological mechanisms and fit to time courses of clinical variables. This methodology can facilitate the exploration of inflammatory mediator interactions, indicators of patient prognosis, and therapeutic influences.

In the current study, we derived an ODE model that incorporates information about cerebrospinal fluid (CSF) biomarkers and their role in regulating both microglial behavior and subsequent secondary tissue damage. We present model fits to clinical CSF cytokine data, and qualitative projections of microglial and tissue damage states. From these results, we derived ensembles of model parameters associated with distinct patient clusters with favorable and unfavorable scores on the Glasgow Outcome Scale (GOS) at 6 months post-injury. We propose this modeling framework as a tool to suggest differences in underlying mechanisms that potentially contribute to the temporal diversification of acute neuroinflammatory patterns after injury. We demonstrate these mechanistic computational modeling results as proof-of-concept data that can provide key qualitative insights, to be complemented by subsequent experimental testing of model predictions, toward the design of personalized intervention strategies for TBI.

## Materials and methods

### Study protocol/TBI participants

This study was approved by the Institutional Review Board at the University of Pittsburgh. We utilized clinical, demographic, and CSF inflammatory biomarker data from *n* = 89 individuals with severe TBI who were between 16 and 75 years of age and had admission Glasgow Coma Scale (GCS) scores ≤ 8 with positive findings on head computed tomography. Participants were excluded under the following circumstances: a penetrating TBI, documented prolonged cardiac/respiratory arrest at the time of injury, evidence of brain death in the first 3 days of injury, or an Abbreviated Injury Scale (AIS) score ≥ 5 in a non-head region. Based on International Classification of Disease (ICD)-9 codes reported at the time of acute care discharge, no individuals in this study had a history of or concurrent malignant neoplasms (implicating cancer) at the time of injury. Individuals with TBI received care consistent with *The Guidelines for the Management of Severe Head Injury* [18].

### CSF sample collection and processing

CSF samples (*n* = 567) were collected up to twice daily via extraventricular drain, as close to the hours of 7 AM and 7 PM as possible, for up to 5 days after injury as a part of routine care. In some instances, it was not possible to obtain CSF samples due to conflicts with clinical care, minimal CSF output, or removal from the intensive care unit (ICU).

Neuroinflammatory markers were measured using Luminex™ bead array assays (Millipore, Billerica, MA). These markers included interleukin (IL)-1β, IL-4, IL-5, IL-6, IL-7, IL-8, IL-10, IL-12, tumor necrosis factor alpha (TNF-α), soluble vascular adhesion molecule-1 (sVCAM-1), soluble intracellular adhesion molecule-1 (sICAM-1), and soluble Fas (sFAS). All inflammatory markers were considered in principal component and cluster analyses, as reported previously [19]. However, only a subset of these markers (IL-1β, IL-4, IL-10, and IL-12) was utilized in the mathematical model to represent hallmark pro- and anti-inflammatory mediators of microglia activity and brain tissue integrity [2, 20].

### Demographic and clinical variables

Demographic and clinical variables were collected via in-person interview and electronic medical record abstraction. The variables reported include age, sex, body mass index (BMI), injury severity scale (ISS) score, best GCS score in 24 h, and length of stay in acute care. The functional capacity of patients was assessed using the GOS at 6 months to measure long-term global recovery. Individuals received the following GOS scores accordingly: (1) dead, (2) vegetative state, (3) severe disability, (5) moderate disability, and (5) good recovery [21].

### Statistical analysis

Patient demographic, clinical, and inflammatory data were statistically analyzed using SAS version 9.4 (SAS Institute Inc., Cary, NC). Descriptive measures included mean, median, standard error of the mean (SE), and interquartile range (IQR) for continuous variables and percentages for categorical variables. Bhattacharyya statistics were calculated using STATA version 14 (StataCorp, College Station, TX). Statistical significance was set as *p* < 0.05 in this study.

### Principal component and cluster analysis

Principal component analysis (PCA) previously reported by R.G. Kumar et al. identified sets of inflammatory markers that contribute the greatest variance to acute CSF inflammatory profiles (days 0–5 post-TBI) [19]. Individuals were assigned a score for each significant principal component (PC) based on their levels of the inflammatory markers that contribute to that particular PC. A non-hierarchical *k*-means cluster analysis was then conducted on the scores for significant PCs for all individuals, characterizing patient subpopulations with similar acute neuroinflammatory profiles post-TBI. This analysis yielded two major cluster groups that were distinguished by distinct CSF inflammatory profiles for days 0–3 post-TBI. Within clusters 1 and 2 identified by R.G. Kumar et al. [19], we grouped individuals in each cluster based on GOS scores. Unfavorable and favorable outcome groups were defined as individuals exhibiting a 6-month GOS = 1,2,3 (dead/vegetative state/severe disability) or a 6-month GOS = 4,5 (moderate disability/good recovery), respectively. These clustering techniques produced the following patient groups: an unfavorable outcome group with a relatively high inflammatory load (cluster 1), a favorable outcome group with a lower inflammatory load (cluster 2A), and an unfavorable outcome group with a similar lower load (cluster 2B).

### Ordinary differential equation model development

We built upon the initial statistical work described above by R.G. Kumar et al. [19], which identified patient subgroups with distinct acute neuroinflammatory profiles, by using mathematical modeling techniques to investigate potential mechanisms in post-TBI neuroinflammation that may underlie the observed patient heterogeneity. A system of ODEs was derived to represent the temporal dynamics of four cytokines (IL-1β, IL-4, IL-10, and IL-12), which were determined from the literature to constitute the minimal set needed to represent the range of roles played by cytokines in regulating both microglial responses and subsequent secondary CNS tissue damage time courses [2, 20]. The equations consist of terms that represent biological processes—such as production, inhibition, saturation, or decay of particular inflammatory mediators—and capture changes in those inflammatory mediator levels over time. ODE models provide a framework for representing multiple interactions and dependencies between mediators post-injury.

**Fig. 1:**
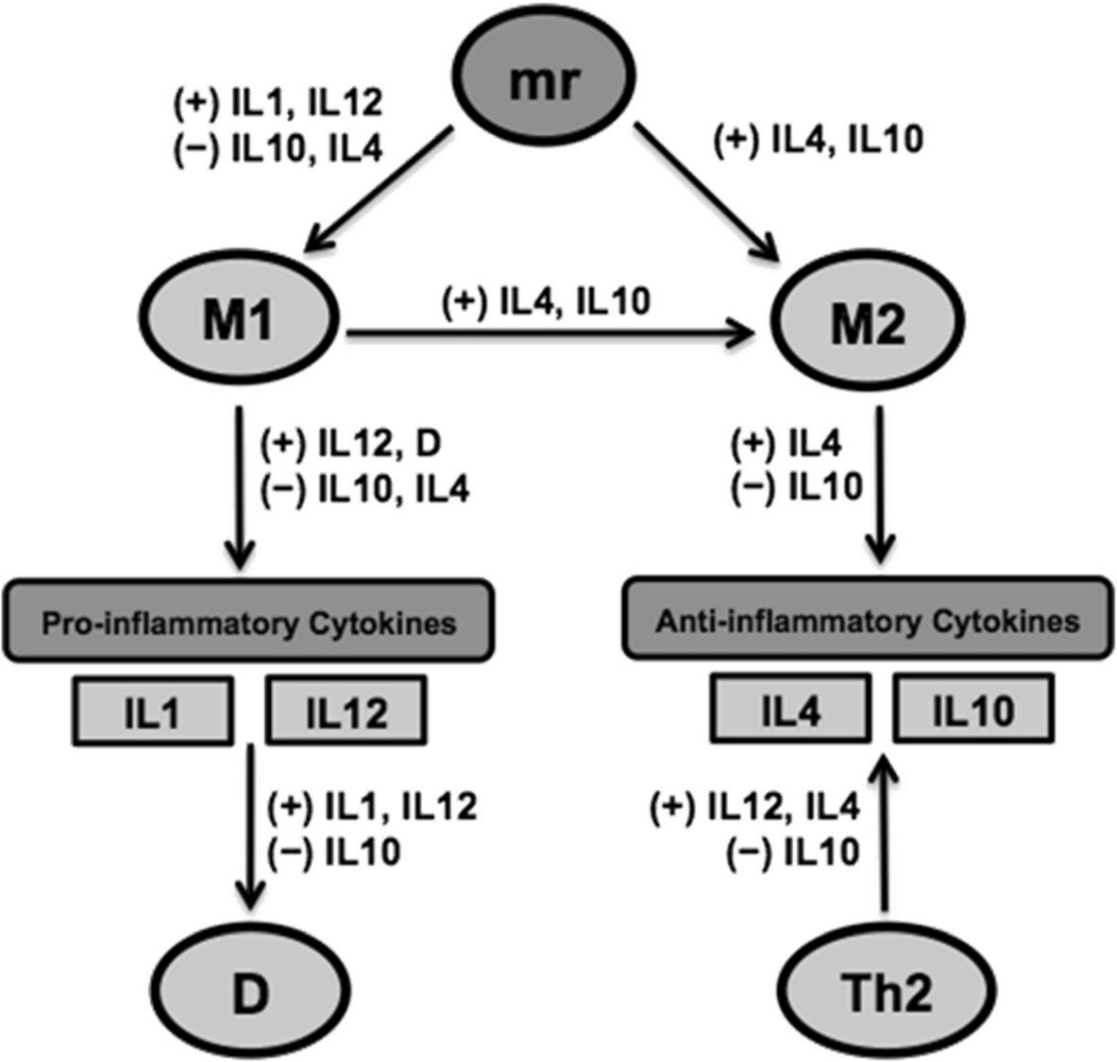
TBI acute neuroinflammation network schematic. Model variables include resting microglia (*mr*), M1-like microglia (*M*1), M2-like microglia (*M*2), interleukin(IL)-1 (*IL*1), IL-12 (*IL*12), IL-4 (*IL*4), IL-10 (*IL*10), tissue damage (*D*), and type 2 T-helper cells (*Th*2). Model components appearing next to pathways stimulate (+) or inhibit (−) the correspoding reaction

To guide the derivation of the ODE model, a simplified conceptualization of microglial contributions to acute neuroinflammation post-TBI was formed (Fig. 1). This theoretical model was limited to a core set of mediators previously identified as having distinct roles in microglial behavior. The corresponding reduced mathematical model, much in the spirit of previous reduced ODE models that have been proven to be useful in the analysis of acute inflammatory responses [13–17], aimed to characterize acute neuroinflammatory and microglial dynamics through seven differential equations for the following biological variables: M1-like microglia (*M*1), M2-like microglia (*M*2), IL-1β (*IL*1), IL-12 (*IL*12), IL-10 (*IL*10), IL-4 (*IL*4), and secondary tissue damage (*D*). The differential equations include 52 parameters with direct biological interpretations, as shown in Table 1. The following sections detail the acute neuroinflammatory processes post-TBI that are encompassed within our ODE model and a priori information that guided its derivation.

**Table 1:** Biological interpretations of ODE model parameters for acute neuroinflammation

Parameter	Description
Resting microglia	
*s*_*mr*_	Source of resting microglia (*mr*)
*μ*_*mr*_	Decay rate of *mr*
*M*1-like activation	
*k*_*n1*_	Rate of *M*1 activation by *IL*1
*k*_*n12*_	Rate of *M*1 activation by *IL*12
*b*_*n*_	Half-activation constant
*x*_*n*_	Hill coefficient
*a*_*∞1*_	Threshold-like factor for *IL*4 and *IL*10 inhibition of *M*1 polarization
*M*2-like activation	
*k*_*n4*_	Rate of *M*2 activation by *IL*4
*k*_*n10*_	Rate of *M*2 activation by *IL*10
*y*_*n*_	Half-activation constant
*z*_*n*_	Hill coefficient
Microglial phenotype switch	
*τ*_*n4*_	Relative effectiveness of *IL*4 in driving *M*1 to *M*2 differentiation
*τ*_*n10*_	Relative effectiveness of *IL*10 in driving *M*1 to *M*2 differentiation
*m*_*n*_	Half-activation constant
*g*_*n*_	Hill coefficient
Microglial decay	
*μ*_*M1*_	Decay rate of *M*1
*μ*_*M2*_	Decay rate of *M*2
Cytokine release by Th2 cells	
*k*_*tbase*_	Baseline rate of *Th2* cytokine release
*k*_*tn12*_	Relative effectiveness of *IL*12 in inhibiting the *Th1* pro-inflammatory response and promoting *Th2* anti-inflammatory response
*r*_*n*_	Half-activation constant
*c*_*n*_	Hill coefficient
Pro-inflammatory cytokines	
*k*_*M1base*_	Baseline rate of *M*1 pro-inflammatory cytokine release
*kcd*	Relative effectiveness of tissue damage (*D*) in promoting pro-inflammatory cytokine production
*v*_*n*_	Half-activation constant
*h*_*n*_	Hill coefficient
*k*_*pn1*_	Relative rate of *IL*1 release
*k*_*pn12*_	Relative rate of *IL*12 release
*μ*_*n1*_	Decay rate of *IL*1
*μ*_*n12*_	Decay rate of *IL*12
Anti-inflammatory cytokines	
*k*_*M2base*_	Baseline rate of *M*2 anti-inflammatory cytokine release
*kc4*	Relative effectiveness of *IL*4 in promoting anti-inflammatory cytokine production
*w*_*n*_	Half-activation constant
*q*_*n*_	Hill coefficient
*a*_*∞2*_	Threshold-like factor for *IL*10 inhibition of cytokine release
*k*_*tn10*_	Rate of *IL*10 release by *Th2* cells
*k*_*tn4*_	Rate of IL4 release by *Th2* cells
*k*_*pn10*_	Rate of IL10 release by *M*2
*k*_*pn4*_	Rate of IL4 release by *M*2
*μ*_*n10*_	Decay rate of *IL*10
*μ*_*n4*_	Decay rate of *IL*4
Tissue damage	
*α*_*n12*_	Rate of *IL*12 induction of damage
*α*_*n1*_	Rate of *IL*1 induction of damage
*γ*_*M1*_	Rate of damage clearance by *M*1
*γ*_*M2*_	Rate of damage clearance by *M*2
*r*_*M1*_	Rate of damage production by *M*1

#### Microglial activation from resting state

Since the contemporary concept of resting microglial activation to a classic pro-inflammatory M1-like or an alternative anti-inflammatory M2-like state is still evolving, our model focuses on influences from the CNS cytokine microenvironment that likely affect microglial dynamics following TBI. Resting microglia (*mr*), the brain’s resident immune cells, continually sample the local microenvironment, surveying for any deviation from homeostasis [22, 23]. Following TBI, resting microglia polarize into two broad activation states, M1- or M2-like, in response to early cellular mediators released by injured cells [23, 24].

The terms *Rm*1 and *Rm*2 below describe the cytokine-based cues for the activation of resting microglia into M1- and M2-like microglia, respectively. The numerator in the equation for *Rm*1 is a mathematical expression known as a Hill function that tends to a constant value as *IL*1 and *IL*12 become large. This term represents the saturating promotion of M1 microglial polarization by the initiator molecule IL-1β and pro-inflammatory molecule IL-12. Both the denominator of the *Rm*1 expression and the full *Rm*2 expression are based on the action of anti-inflammatory agents, IL-10 and IL-4, which accumulate at the site of injury, limiting further M1 polarization and driving microglia differentiation to the M2 state:

$$ Rm1=\frac{\frac{{\left({k}_{n1}\bullet IL1+{k}_{n12}\bullet IL12\right)}^{x_n}}{b_n^{x_n}+{\left({k}_{n1}\bullet IL1+{k}_{n12}\bullet IL12\right)}^{x_n}}}{1+{\left(\frac{IL10+ IL4}{a_{\infty 1}}\right)}^2} $$

$$ Rm2=\frac{{\left({k}_{n4}\bullet IL4+{k}_{n10}\bullet IL10\right)}^{z_n}}{y_n^{z_n}+{\left({k}_{n4}\bullet IL4+{k}_{n10}\bullet IL10\right)}^{z_n}}. $$

Together, these terms shape how the brain’s resting microglia reserve is allocated to undergo M1- or M2-like activation based on cytokine cues from the local environment [23, 25, 26]. For modeling purposes, we assume that resting microglia (*mr*) are produced at a constant rate *s*_*mr*_ and decay at a constant rate μ_mr_*,* and that the overall level of activation rapidly equilibrates to changes in *Rm*1 and *Rm*2. That is, we set to zero the right-hand side of the ODE:

1 $$ \frac{dmr}{dt}={s}_{mr}- Rm1\bullet mr- Rm2\bullet mr-{\mu}_{mr}\bullet mr $$

and solve for *mr* to derive the quasi-steady-state expression:

2 $$ mr\approx \frac{s_{mr}}{Rm1+ Rm2+{\mu}_{mr}}. $$

#### Microglial polarization dynamics

Although recent conceptual reviews have suggested that there may be an activation spectrum of microglial states [2, 9, 10, 22, 23], for simplicity we consider the two extremes of characteristic M1 and M2 states. In addition to the activation of resting microglia described in “Microglial activation from resting state” subsection, and basic decay or cell death rates, levels of microglia in these states are influenced by the repertoire of neuroinflammatory reactions occurring post-TBI. These processes cause cytokine levels to fluctuate and, in turn, drive microglia phenotype switching [22, 27]. We introduce a Hill function *Rms* to represent contributions of anti-inflammatory mediation by IL-4 and IL-10 toward shifting neurotoxic M1-like microglia into a neuroprotective and reparative M2-like microglia state:

$$ Rms=\frac{{\left({\tau}_{n4}\bullet IL4+{\tau}_{n10}\bullet IL10\right)}^{g_n}}{m_n^{g_n}+{\left({\tau}_{n4}\bullet IL4+{\tau}_{n10}\bullet IL10\right)}^{g_n}}. $$

The majority of cells maintain M2-like status once cellular debris has been cleared from the initial injury and regenerative processes are initiated; therefore, we do not include switching from the M2 to the M1 phenotype. The equations describing the M1- and M2-like microglia population changes thus take the form:

3 $$ \frac{dM1}{dt}= Rm1\bullet mr- Rms\bullet M1-{\mu}_{M1}\bullet M1 $$

4 $$ \frac{dM2}{dt}= Rm2\bullet mr+ Rms\bullet M1-{\mu}_{M2}\bullet M2. $$

#### Pro-inflammatory processes

M1-like microglia are the major agents of pro-inflammatory cytokine secretion in the model, with a release rate that we describe using a term *Rp*. In addition to a baseline level of pro-inflammatory cytokine release, this term includes the promotion of M1 pro-inflammatory cytokine secretion by IL-12. Also included in the numerator of *Rp* is the effect of damaged tissue, with level *D*, which releases damage-associated molecular patterns (DAMPs) that augment pro-inflammatory pathways and recruit other immune cells. Anti-inflammatory mediators, IL-10 and IL-4, act as inhibitors of this positive feedback loop between pro-inflammatory cytokines and delayed tissue damage [27, 28], as represented in the denominator of *Rp*, which overall takes the form:

$$ Rp=\frac{k_{\mathrm{M}1\mathrm{base}}\bullet M1+M1\left(\frac{{\left( IL12+ kcd\bullet D\right)}^{h_n}}{v_n^{h_n}+{\left( IL12+ kcd\bullet D\right)}^{h_n}}\right)}{1+{\left(\frac{IL10+ IL4}{a_{\infty 1}}\right)}^2}. $$

The release rates of pro-inflammatory cytokines, IL-1β and IL-12, by M1 as described in Eqs. (5)–(6) are both dependent on this pro-inflammatory cytokine production term (*Rp*), each modulated by its own scaling factor:

5 $$ \frac{dIL1}{dt}={k}_{pn1}\bullet Rp-{\mu}_{n1}\bullet IL1 $$

6 $$ \frac{dIL12}{dt}={k}_{pn12}\bullet Rp-{\mu}_{n12}\bullet IL12 $$

#### T-helper cell involvement

The proposed ODE model is microglia-based; however, T-helper (Th) cells have considerable influence on neuroinflammatory cascades post-TBI because they synthesize and secrete cytokines relevant to microglial activation and to the M1 → M2 phenotype transition [29]. Therefore, a term *Rt* was designated to account for indirect influences of Type 2 Th (*Th*2) cells on the production of IL-4 and IL-10:

$$ Rt=\frac{k_{tbase}+\left(\frac{{\left( IL4+{k}_{tn12}\bullet IL12\right)}^{c_n}}{r_n^{c_n}+{\left( IL4+{k}_{tn12}\bullet IL12\right)}^{c_n}}\right)}{1+{\left(\frac{IL10}{a_{\infty 2}}\right)}^2}. $$

These *Th*2 cell interactions are crucial in resolving the pro-inflammatory M1-like state by allowing for transition to anti-inflammatory M2-like conditions. As pro-inflammatory IL-12 accumulates in the local environment, *Th*2 cells are signaled to produce IL-4, which initiates M1 polarization to M2 [22] as captured in Eqs. (3)–(4). Another pertinent aspect of these reactions is the self-regulatory nature of IL-10, which is produced by *Th*2 in the presence of IL-4. IL-10 suppresses T cell responses (represented mathematically by its appearance in the denominator of *Rt*) and, in turn, its own production [30]. This autocrine inhibitory signaling protects cells against unregulated anti-inflammatory processes in the return to health.

#### Anti-inflammatory processes

The rate of synthesis and secretion of anti-inflammatory cytokines by M2-like microglia is represented by the term *Ra*. IL-4 serves the dual role of driving additional microglial polarization toward the M2 phenotype and inducing anti-inflammatory cytokine production [28]. Negative feedback by IL-10 (as described in “T-helper cell involvement” subsection) is crucial to the resolution of cytokine production [30], and is thus represented in the denominator of the anti-inflammatory cytokine production term:

$$ Ra=\frac{k_{\mathrm{M}2\mathrm{base}}\bullet M2+M2\left(\frac{{\left( kc4\bullet IL4\right)}^{q_n}}{w_n^{q_n}+{\left( kc4\bullet IL4\right)}^{q_n}}\right)}{1+{\left(\frac{IL10}{a_{\infty 2}}\right)}^2}. $$

*Th*2 and *M*2 contributions to anti-inflammatory cytokine production, together with baseline decay of cytokines, are combined to give the following ODEs:

7 $$ \frac{dIL10}{dt}={k}_{tn10}\bullet Rt+{k}_{pn10}\bullet Ra-{\mu}_{n10}\bullet IL10 $$

8 $$ \frac{dIL4}{dt}={k}_{tn4}\bullet Rt+{k}_{pn4}\bullet Ra-{\mu}_{n4}\bullet IL4. $$

#### Secondary tissue damage

While the acute neuroinflammatory response is designed to be neuroprotective, certain processes cause adverse secondary injury reactions, particularly if they are sustained or exaggerated [22, 31]. If unregulated, M1-like microglia can compromise healthy tissue through unselective phagocytosis and lead to progressive neurodegeneration. Also, pro-inflammatory positive-feedback loops may induce a prolonged, detrimental inflammatory cycle in which inflammatory processes overwhelm anti-inflammatory constraints. The potentially damaging effects of pro-inflammatory cytokines lie in their promotion of additional pro-inflammatory pathways, as opposed to direct tissue damage [14, 23].

In our model, damage (*D*), which evolves according to the ODE (9), represents a qualitative secondary tissue damage level, serving as a proxy for long-term outcome among individuals with TBI. Equation (9) incorporates factors that contribute to further neurodegeneration including exacerbated pro-inflammatory pathways by IL-1β and IL-12, which are inhibited by IL-10, and M1-like microglia that release neurotoxic chemicals and may unselectively phagocytize healthy tissue [32, 33]. Additionally, neuroprotective features of microglia are captured in Eq. (9) to represent processes that mitigate further tissue damage. M1-like microglia are essential to tissue recovery via their role in host defense mechanisms (pro-inflammatory cytokine, chemokine, and reactive oxygen species release) that recruit immune cells to the site of injury, antigen-presenting capabilities, and limited phagocytic activity [22, 33]. M2-like microglia act to alleviate damage by clearing dysfunctional neurons and cellular debris, promoting neurogenesis and remyelination, as well as suppressing destructive inflammatory processes [34, 35], as represented in the negative terms in Eq. (9):

9 $$ \frac{dD}{dt}=\frac{\alpha_{n12}\bullet IL12+{\alpha}_{n1}\bullet IL1}{1+{\left(\frac{IL10}{a_{\infty 2}}\right)}^2}+{r}_{M1}\bullet M1-{\gamma}_{M1}\bullet M1\bullet D-{\gamma}_{M2}\bullet M2\bullet D. $$

### Parameter optimization

Our ODE model (Eqs. (3)–(9)) includes a large number (52) of parameter values, mostly representing rate constants, half-activation, or saturation levels, and exponents that affect sensitivity to changes in levels of evolving quantities. These parameter values were constrained by previous literature and biological requirements (e.g., positivity), but many have not been measured experimentally. Our approach was to tune parameter values through parameter optimization methods to produce outputs consistent with observed clinical inflammatory biomarker trajectories while remaining within the constraints that we imposed. Optimization was performed over the 45 parameters listed in Table 1, as well as over initial condition values for *M*1, *M*2, *IL*1, *IL*12, *IL*10, *IL*4, and *D*. Model integration and parameter optimization were performed using Matlab (MathWorks, Natick, MA).

For each patient group, the model was fit to computed CSF cytokine averages over the first 5 days following TBI in 6-h increments. A moving-average smoothing procedure was applied to the cytokine data to diminish the effect of outliers and short-term fluctuations in the data. Specifically, we defined overlapping 12-h bins, each shifted by 6 h relative to the previous bin, consisting of data from 0 to 12, 6–18, 12–24 h post-TBI, and so on. For each cytokine, all values that occurred during the time range encompassed by a bin were averaged together.

Initial parameter estimates were guided by available values reported in the literature [14, 16]. The Nelder-Mead simplex method was then employed as a nonlinear optimization algorithm to determine sets of initial conditions and parameter values that best fit model outputs, obtained by numerical integration of Eqs. (3)–(9) from a given set of initial conditions, to the averaged clinical data [36]. This method was implemented by using the *fminsearch* function in Matlab to attain optimal model fits to cytokine data from each subcluster. The Matlab function *ode15s* was used to solve the system of ODEs. Goodness of fit to patient data was determined by evaluating an error function that compared the model-generated cytokine values to mean patient cytokine values at every 6-h mark, summing the squared differences and dividing by the squared standard deviation to normalize. Additionally, the following microglia heuristics were set in place: *M*1 > *M*2 prior to day 2, *M*2 > *M*1 after day 3, and *M*1, *M*2 > 2 for the entire time course. A penalty value of 100 was added to the error calculation for each of these conditions that was violated. Empirically, smaller penalty values did not yield enforcement of the desired conditions.

### Ensemble of optimal model fits

Parameter estimation techniques led to a representative parameter set that fit cytokine data for each patient group. This baseline parameter set was then randomly perturbed to provide new parameter sets that would serve as starting estimates for an additional 100 iterations of subsequent parameter estimation. Perturbations were randomly selected from a uniform distribution from 0.5 to 1.5 times the baseline parameter set values. The parameter optimization procedure was repeated for each of the 100 distinct parameter sets for each patient group, yielding an ensemble of model fits. The use of this method was intended to allow for the possibility that diverse parameter sets could provide similar model fits and to capture the variability in biological characteristics that would naturally occur across a patient population.

### Parameter distribution analyses

The ensembles of 100 model trajectories and parameter sets from which they were generated were compared across patient groups to investigate relative differences in cytokine, microglia, and tissue damage temporal dynamics. Statistically comparing the distributions of each parameter value between clusters was used to elucidate physiological mechanistic differences that potentially explain divergent clinical outcomes post-TBI.

#### Bhattacharyya metrics

The Bhattacharyya distance (BD) was calculated as a statistical comparison of parameter distributions between patient groups. BD quantifies the level of spread between distributions by incorporating their means and variances. The Bhattacharyya coefficient (BC) measures the degree of overlap between distributions [37]. A BC of 0 indicates that the parameter distributions are non-overlapping, while larger values signify greater similarity. Conversely, a greater BD is associated with greater spread between parameter distributions. These metrics were computed via the *bhatt* function in STATA.

#### Sensitivity analysis

In differential equation modeling, the values of particular parameters may influence model output at certain time points more than others. Among parameter distributions that differed significantly in the previous statistical analyses, those that exhibit most influence on model behavior merit the most attention as potential sources of cluster differences in acute neuroinflammatory mechanisms post-TBI.

Sensitivity analyses were conducted in Matlab by perturbing each parameter value individually by ± 2% and observing subsequent changes in model output. Model sensitivity (*S*) is calculated as:

$$ S=\frac{\Delta  x(t)}{\Delta  p}\bullet \frac{p}{x(t)}, $$

where *∆x*(*t*) is the difference in model outputs at time *t*, and *∆p* is the difference between the perturbed and original parameter value. The model sensitivity is normalized to account for the nominal value of the parameter *p* and model output *x*(*t*).

## Results

### Acute neuroinflammatory profile association with long-term clinical outcome

In previous work, R.G. Kumar et al. identified groups of patients with similar neuroinflammatory profiles in the acute phase with no a priori knowledge of long-term outcome [18]. However, further analysis revealed significant associations between cluster group assignment and long-term outcome based on GOS score. The vast majority (93.1%) of individuals in cluster 1 experienced unfavorable outcomes (GOS = 1,2,3), whereas members of cluster 2 varied greatly in their recovery status 6 months post-TBI. We thus grouped cluster 2 into cluster 2A, consisting of the 45.5% of individuals in cluster 2 reporting favorable outcomes (GOS = 4,5), and cluster 2B, composed of the remaining 54.5% with unfavorable outcomes (GOS = 1,2,3). Grouping by 6-month GOS score was not implemented in cluster 1 due to the small number of favorable outcome patients belonging to this group (*n* = 2). GOS scores at 6 months post-TBI (Table 2) and clinical characteristics (Table 3) are reported for each of these patient clusters. Those in cluster 1 were substantially older compared to individuals in cluster 2, and hospital length of stay was shorter, a finding likely due (in part) to higher acute mortality rates in this patient cluster. These groupings show that (1) different acute neuroinflammatory response profiles may lead to similar outcomes, and (2) similar acute neuroinflammatory responses may lead to disparate outcomes.

**Table 2:** Six-month Glasgow Outcome Scale score by cluster group

6-mo. GOS score, *n* (%)	Cluster 1	Cluster 2A	Cluster 2B
1	14 (48.28)		11 (34.38)
2–3	13 (44.83)		21 (65.63)
4–5	2 (6.90)	28 (100)

**Table 3:** Clinical and demographic associations with cluster group

	Cluster 1	Cluster 2A	Cluster 2B	*p* value
	(*n* = 29)	(*n* = 28)	(*n* = 32)	
Age, Mean (SE)	*46*.*09* (*3*.*26*)	*31*.*29* (*2*.*70*)	*32*.*59* (*2*.*64*)	*0*.*0026*
Sex, Men (%)	23 (71.88)	25 (89.29)	27 (84.38)	0.1949
BMI, Mean (SE)	26.14 (0.90)	27.71 (1.06)	26.99 (1.15)	0.5391
ISS score, Mean (SE)	32.81 (1.72)	33.30 (1.27)	34.53 (1.50)	0.5804
GCS score (best in 24 h.), Median (IQR)	6 (5–7)	7 (6–9.25)	6.5 (5–7)	0.1703
Length of stay in acute care (days), Mean (SE)	*17*.*49* (*1*.*77*)	*21*.*68* (*1*.*64*)	*25*.*09* (*2*.*21*)	*0*.*0088*

Italic signifies statistical significance at α = 0.05

### ODE model trajectories obtained by fitting to CSF cytokine time courses

**Fig. 2:**
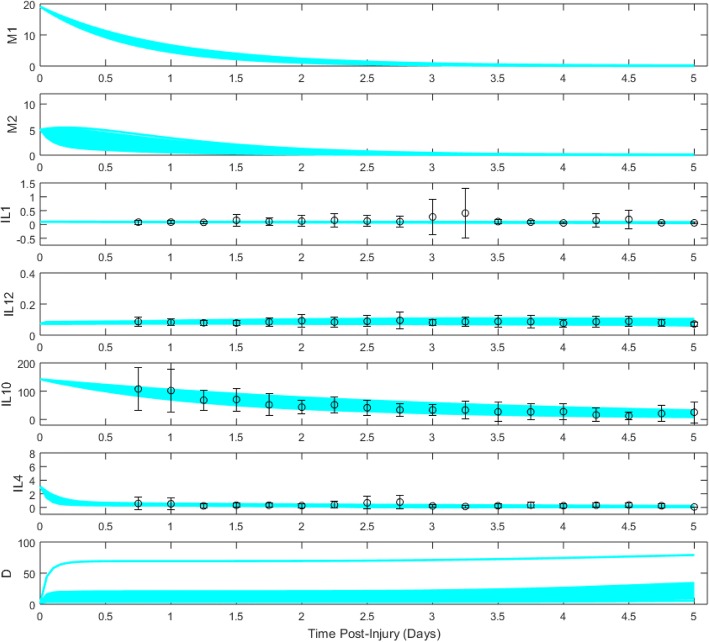
Cluster 1 ensemble of model trajectories for days 0–5 post-TBI. Dots represent moving-average data (see “Parameter optimization” section) collected from patients, while bars represent standard error of the mean

**Fig. 3:**
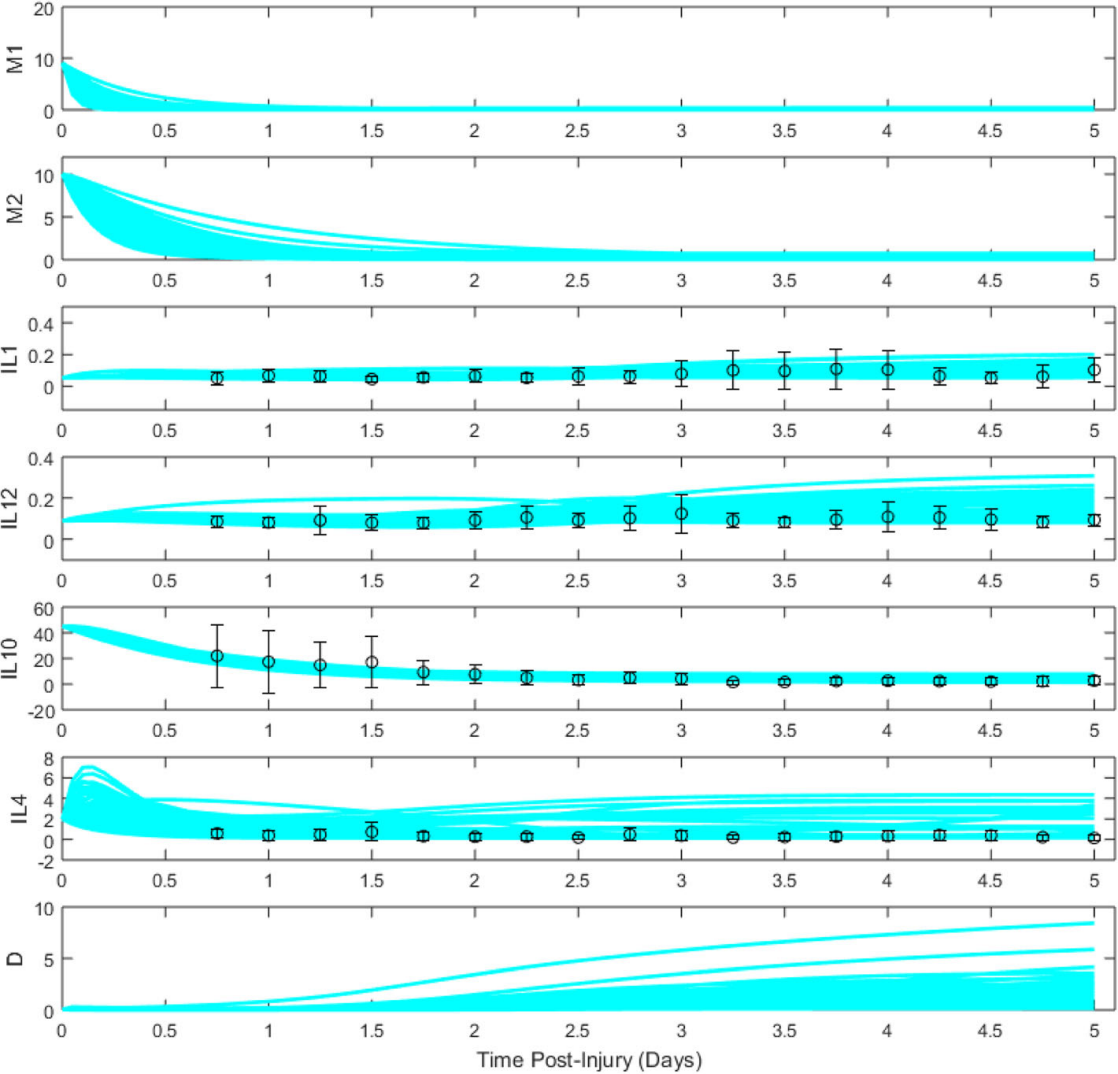
Cluster 2A ensemble of model trajectories for days 0–5 post-TBI. Dots represent moving-average data (see “Parameter optimization” section) collected from patients, while bars represent standard error of the mean

**Fig. 4:**
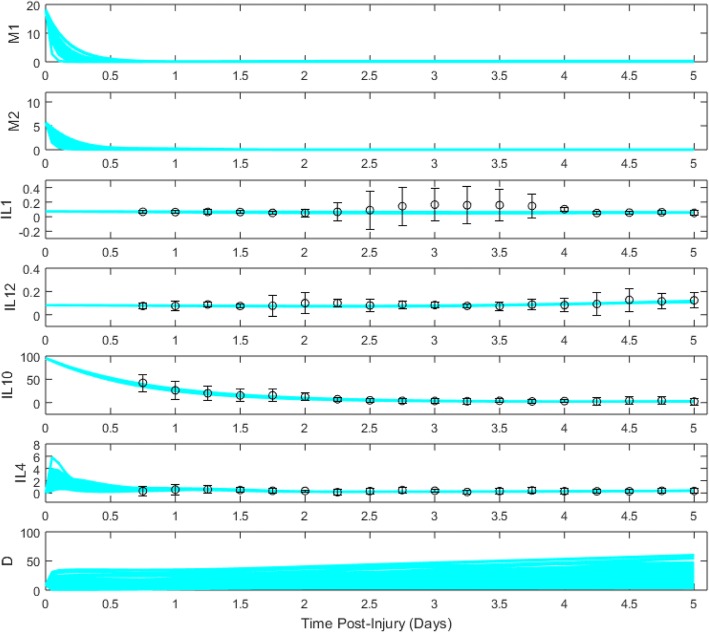
Cluster 2B ensemble of model trajectories for days 0–5 post-TBI. Dots represent moving-average data (see “Parameter optimization” section) collected from patients, while bars represent standard error of the mean

An ODE model (Eqs. (3)–(9)) for the combined temporal evolution of M1- and M2-like microglia, levels of cytokines IL-1β, IL-12, IL-10, IL-4, and a secondary tissue damage variable *D* was derived based on well-supported biology underlying the acute inflammatory response to TBI (“Ordinary differential equation model development” section). Parameter optimization methods were used to find collections of the 52 model parameters for which model trajectories best fit the averaged and smoothed data for each patient group (“Parameter optimization” section). Ensembles of 100 model fits, which were produced by a range of initial parameter sets along with averaged cytokine data values, are shown for each patient cluster in Figs. 2, 3, and 4.

Divergent responses are observable among the model trajectories for microglia, cytokines, and tissue damage across the three patient clusters. Cluster 1, which had the highest inflammatory load associated with unfavorable outcomes, demonstrated the most prolonged and elevated microglial expression of the three clusters. Cluster 1 trajectories were also characterized by relatively constant levels of pro-inflammatory cytokines IL-1β and IL-12, high initial values of IL-10, and rapid decay of IL-4. Interestingly, two types of damage responses were observed: a more common response in which damage rose only slowly, and a minority response with a rapid rise in damage following by a slow increase, a finding that likely captures heterogeneity expected in any patient population.

Trajectories for cluster 2A, which was associated with favorable patient outcomes, showed a more rapid microglial decay than cluster 1, with a noticeably faster decay of M1-like than M2-like microglia. Somewhat surprisingly, levels of pro-inflammatory cytokines IL-1β and IL-12 grew over time, with higher IL-12 levels than were observed for cluster 1. On the anti-inflammatory side, IL-10 levels were lower in cluster 2A compared to cluster 1, while IL-4 levels were slower-decaying than for cluster 1, and in some cases even increased, presumably helping to suppress *D* values.

Model projections for cluster 2B, characterized by lower inflammatory loads than cluster 1 yet unfavorable outcomes, showed the most elevated levels of tissue damage and the most transient microglial response of the three clusters. Additional features of cluster 2B trajectories included a significant rise in IL-12 levels near the end of the simulations, IL-10 levels that started high but decayed rapidly, and an abrupt decay of IL-4. These factors suggest that there may be a significant pro-inflammatory contribution which is exacerbated by transient anti-inflammation to the high levels of *D* arising for cluster 2B.

### Comparison of parameter distributions between clusters

Optimal model fits to patient data were achieved by tuning biological parameters that appear in our set of differential equations (Eqs. (3)–(9)) describing the time course of acute neuroinflammation post-TBI. The resulting distributions of values for each parameter were compared to identify those parameters with the most significant variation across patient clusters (“Parameter distribution analyses” section). Each model parameter has a corresponding biological interpretation (Table 1), and the neuroinflammatory role of each identified parameter was considered in order to understand its contribution to the relative temporal dynamics of cytokines, microglia, and tissue damage that differentiate long-term outcome. Table 4 shows a summary of the most dissimilar parameter distributions between clusters, based on Bhattacharyya metrics of overlap (BC) and spread (BD)**.** Parameter distribution differences were ranked by ascending overlap (BC) and descending spread (BD). We report and discuss the most disparate parameter distributions that were selected under the following criterion: *BC* ≤ 0.38, and *BD* ≥ 0.97.

**Table 4:** Sensitive parameters with most disparate distributions between each cluster pair

Parameter	Bhattacharyya Metrics	
	*BC*	*BD*
Cluster 1 vs. Cluster 2A		
*a*_*∞*2_	0	n/a
*k*_*pn*12_	0	n/a
*μ*_*M*1_	0	n/a
*μ*_*n*10_	0	n/a
*k*_*pn*1_	0.2	1.61
Cluster 1 vs. Cluster 2B		
*μ*_*M*1_	0	n/a
*μ*_*n*10_	0	n/a
*k*_*pn*12_	0.01	n/a
*h*_*n*_	0.134	2.01
*k*_*pn*1_	0.2	1.61
*μ*_*M*2_	0.209	1.56
*μ*_*n*12_	0.245	1.41
Cluster 2A vs. Cluster 2B		
*a*_*∞*2_	0	n/a
*μ*_*M*2_	0.045	3.11
*v*_*n*_	0.3	1.2
*k*_*pn*12_	0.379	0.971

Only parameters with model sensitivities exceeding a sensitivity threshold of 2 were included

In conjunction with Bhattacharyya tests applied to compare parameter distributions between clusters, a parameter sensitivity analysis was implemented through Matlab to ensure that the significantly different parameters under consideration also significantly influence model behavior (“Sensitivity analysis” section). Model sensitivities to values of all parameters reported in Table 4 exceeded our sensitivity threshold of 2. An additional file provides the ranges and averages of each dissimilar parameter distribution by pairwise cluster comparisons (see Additional file 1).

#### Cluster 1 vs. cluster 2A

**Fig. 5:**
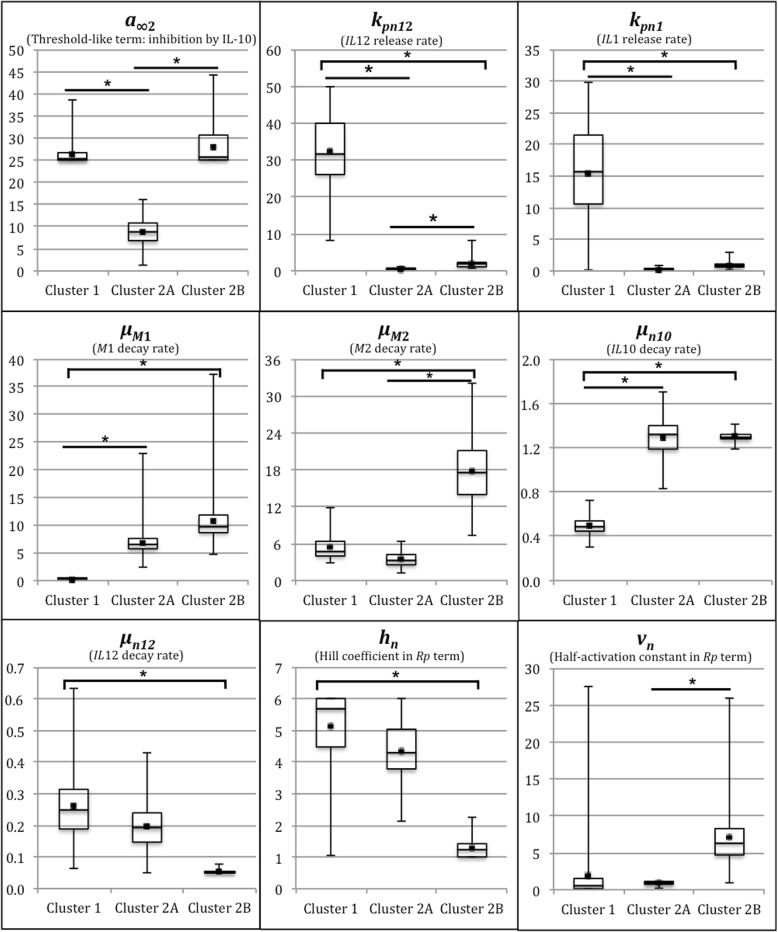
Disparate parameter distributions between patient clusters. Box and whisker plots depict the distributions of the most dissimilar parameter values between clusters 1, 2A, and 2B. Dots represent the parameter value averages by cluster. A star represents statistical significance for pairwise cluster comparisons

Cluster 1 and cluster 2A were the most dissimilar groups in this analysis, differing in acute neuroinflammatory marker levels and 6-month GOS score. Differences in the degree of acute neuroinflammation post-TBI are apparent in the disparate parameter distribution differences shown between clusters in Fig. 5. A more aggressive and sustained course of inflammation is evident for cluster 1 as reflected in greater release rates of IL-1β (*k*_*pn*1_) and IL-12 (*k*_*pn*12_) from activated M1-like microglia and slower decay rates of M1 (*μ*_*M*1_) and IL-10 (*μ*_*n*10_) during acute injury. The level of *a*_*∞*2_ is greater in cluster 1 than in cluster 2A, which points to an ineffectiveness of IL-10 at inhibiting pro-inflammation in cluster 1 patients, also consistent with the elevated inflammatory load in cluster 1.

#### Cluster 1 vs. cluster 2B

While clusters 1 and 2B both reported unfavorable outcomes at 6 months, individuals in these clusters expressed different acute neuroinflammatory loads. Figure 5 depicts the most dissimilar parameter distributions between these clusters, which potentially explain maladaptive features of the inflammatory response in each cluster. Cluster 2B exhibits a more transient inflammatory response with greater decay rates of M1- (*μ*_*M1*_) and M2-like (*μ*_*M2*_) microglia, as well as IL-10 (*μ*_*n10*_). This finding suggests that the acute inflammatory profile of cluster 2B individuals does not sustain microglial signaling and anti-inflammatory control for an adequate amount of time to achieve an effective, balanced inflammatory response.

In contrast, cluster 1 demonstrates a stronger early pro-inflammatory response driven by elevated production of IL-1β (*k*_*pn*1_) and IL-12 (*k*_*pn*12_) by *M*1, with greater sensitivity to accumulating IL-12 and *D* levels (larger *h*_*n*_) leading to exacerbated pro-inflammatory pathways. Negative feedback is also apparent, as mean levels of IL-10 are significantly greater in cluster 1 to counterbalance greater pro-inflammatory cytokine production rates. Despite greater IL-12 production rates by cluster 1 than those observed in cluster 2B, its decay is also more rapid (*μ*_*n*12_), potentially resulting in inadequate signaling to Th2 cells to increase anti-inflammatory cytokine production and counter the excessive pro-inflammatory presence.

#### Cluster 2A vs. cluster 2B

Cluster 2A and 2B, which were initially one inflammatory profile group from previous PCA and cluster analysis, were investigated separately to determine which neuroinflammatory pathways drove differential outcomes at 6 months. Figure 5 highlights dissimilar parameter distributions between these clusters that potentially explain the disparity in their inflammatory programs post-TBI. Once again, the rapid decay of M2 in cluster 2B patients (*μ*_*M*2_) is evident. This effect translated into fast decreases in anti-inflammatory levels, which is coupled with a less effective inhibition of pro-inflammatory effects by IL-10 (*a*_*∞*2_). Cluster 2B also features a greater production of IL-12 than cluster 2A (*k*_*pn*12_). Together, these factors imply that cluster 2B individuals exhibit short-lived, less potent M2 responses yielding neuroinflammatory behavior that does not sufficiently impact the acute tissue damage post-TBI.

### CSF biomarker levels by cluster

R.G. Kumar et al. found considerable differences between clusters 1 and 2 in terms of steroid hormones [18]. Motivated by previous work showing the existence of three patient groups with significantly different cortisol trajectories over the first 6 days post-injury [38], we investigated CSF cortisol level differences between patient groups (Table 5) to supplement our inflammatory marker comparisons. In post-hoc analyses, we found that cluster 1 had higher cortisol levels than both cluster 2A and 2B over the full time course (*p* < 0.001 for all comparisons). For days 0–3, cluster 2A had significantly lower levels of cortisol compared to cluster 2B (*p* = 0.002). However, transitioning into day 4–6, differences in average cortisol levels were no longer significant between cluster 2A and 2B (*p* = 0.340).

**Table 5:** Day 0–3 and 4–6 CSF cortisol levels (ng/mL) by cluster group

CSF Cortisol (ng/mL)	Cluster 1	Cluster 2A	Cluster 2B	*p*-Value
Day 0–3 Mean (SE)	*33*.*34* (*1*.*89*)	*17*.*7* (*1*.*19*)	*22*.*58* (*1*.*68*)	< *0*.*001*
Day 4–6 Mean (SE)	*23*.*07* (*1*.*69*)	*15*.*20* (*1*.*44*)	*15*.*29* (*1*.*44*)	*0*.*006*

Italic signifies statistical significance at α = 0.05

## Discussion

A set of differential equations was derived in this study to model acute neuroinflammatory phenomena following severe TBI, intended to represent dynamic time-dependent interactions within and to generate hypotheses about the complex communication network between resident microglia and neuronal tissue via circulating cytokines. Microglia are potent effector cells in post-TBI neuroinflammation; however, there is limited early clinical information collected regarding their activation, polarization, and functional plasticity. Therefore, we leveraged cytokine dynamics in our model to generate predictions on the state of neuroinflammation, microglia phenotype distributions, and tissue integrity in the acute injury recovery phase (day 0–5). This mechanistic model was generated on a foundation of a priori information regarding core cytokine interactions established empirically in the field of TBI.

Neuroinflammation, although a main contributor to secondary damage post-TBI, is an inherent set of host defense mechanisms aimed to protect and restore tissue integrity [2, 39, 40]. While studies aim to label particular aspects of this response neurotoxic or neuroprotective, this is ultimately a context-dependent consideration. The spatial and temporal regulation of select neuroinflammatory mechanisms may potentially preserve positive physiological function and endogenous tissue homeostatic efforts [3]. Modulating the coordinated balance of pro- and anti-inflammatory cytokines, timely activation of M1 and M2 microglia, and appropriate feedback signaling may provide an adequate amount of pro-inflammation with relatively less secondary tissue damage ensuing [41, 42]. Our ODE modeling techniques provide a platform that recapitulates the relative levels of evolving inflammatory mediators observed with TBI patient data and projects time courses of microglia activation and tissue damage that are mechanistically consistent with these data, thereby predicting the extent to which secondary TBI pathologies may be arising in these clinically observed scenarios.

In this study, we stratified patient subgroups based on long-term neurological outcome from patients that exhibited distinct day 0–3 neuroinflammatory profiles [18]. Leveraging this methodology allowed model fits to be generated for each patient cluster and respective outcome, providing insight on potential differences in their acute neuroinflammatory programs that may contribute to disparate 6-month outcomes.

### Assessing neuroinflammatory status via cytokine trajectories

Initial statistical work by our group applied principal component and cluster analysis to identify variability in acute neuroinflammatory biomarkers among individuals following TBI [18]. These analyses suggested sets of cytokines that demonstrate similar acute expression patterns and may contribute to similar courses of inflammation and tissue recovery, helping to justify the inclusion of a small number of specific cytokines in our current modeling work. In this study, we sought to utilize cytokine dynamics to infer the neuroinflammatory state and subsequent microglial activation profile of each patient cluster.

This methodology is a progressive direction in the TBI field in that relative concentrations of cytokines can be tracked and used to inform hypotheses regarding early injury severity patterns and patient prognosis. While studies have shown that absolute levels of acute anti-inflammatory mediators are predictive measures of initial brain damage and complications, such as intracranial pressure [43], our model illustrates the relative relationships and time courses of mediators to provide a more comprehensive view of neuroinflammation post-TBI. The model was designed on the premise that cytokines are expressed simultaneously following injury, collectively contribute to microglial polarization profiles, and may serve as useful biomarkers patterns to gauge the level of neuroprotection or neurotoxicity in the local microenvironment [3, 41, 44].

### Novelty of modeling TBI-induced neuroinflammation mathematically

In extension to cytokine measurement studies that characterize neuroinflammation post-TBI, this modeling framework is a novel method of investigating (1) temporal dynamics of inflammatory mediators, (2) interdependent cytokine pathways and feedback interactions, and (3) cytokine influences on differential microglia expression and tissue damage responses. Differential equations are well suited for representing post-TBI neuroinflammation because the relative concentration changes of inflammatory mediators can be modeled with respect to time. This initial report serves as a proof-of-concept that implementing mechanistic modeling can further our understanding of inflammatory network dynamics, kinetics, and phenotypic polarization.

While early delivery of anti-inflammatory agents has been an intuitive strategy for containing post-TBI neuroinflammation, the lack of consistent success suggests there is more to consider for this approach. Suppressing a single neuroinflammatory mechanism does not facilitate healthy tissue recovery due to complex interdependencies of neuroinflammation [3, 39]. There has been a shift of perspective in the TBI field that acknowledges not only the dual role of neuroinflammation post-TBI, but also the dichotomy of roles such as perpetuating damage and maintaining homeostasis by individual mediators, including cytokines and microglia phenotypes [9, 45]. The utility of ODE modeling in this context is to elucidate the benefit or detriment of particular mediators relative to time-post-injury and expression of other local mediators.

Model simulations performed on patient-specific data from different outcome groups produced quantitative projections of cytokine dynamics and qualitative predictions of microglia and tissue damage dynamics. The ensembles of parameters, which were tuned to generate optimal fits for each patient cluster, were statistically compared to generate hypotheses regarding differences in the neuroinflammatory regimes of each cluster. These analyses are a contribution to ongoing attempts to characterize the functional roles and heterogeneous effects of microglia and related cytokines in acute TBI neuroinflammation [9, 46]. This report presents the first computational model in the field that aims to model the temporal evolution and M1/M2 phenotypic balance of microglia. In the following sections, we discuss the inflammatory trends, unique parameter differences, and additional clinical considerations for each respective patient cluster and long-term outcome.

### Unfavorable outcome groups (clusters 1 and 2B)—maladaptive features of neuroinflammation

R.G. Kumar et al. had identified a group of individuals (cluster 1) with relatively high day 0–3 CSF inflammatory loads, almost all of which experienced poor long-term outcomes [18]. After subgrouping cluster 2 individuals by 6-month GOS score, we identified another poor outcome group yet with a relatively lower acute inflammatory load (cluster 2B). We hypothesized that the disparity in acute neuroinflammatory profiles could drive differential, yet both detrimental, courses of inflammation post-TBI that hinder recovery.

#### Cluster 1—evidence of prolonged inflammation

Elevated inflammation and highly activated microglia were apparent through several model parameter differences that emerged when comparing across clusters. In comparison to cluster 2B, cluster 1 model parameters were significantly lower for microglial (both M1 and M2 types) and IL-10 decay rates; higher for IL-1β and IL-12 release rates; and higher for sensitivity to pro-inflammatory cytokine and damage signals. This combination of parameter differences is potentially reflective of the failure of acute neuroinflammation and microglia activity to resolve appropriately.

The self-perpetuating cycle of inflammation displayed in cluster 1 trajectories has been shown to be detrimental to recovery for various reasons. While initial upregulation of pro-inflammatory processes is intrinsically a host defense response essential for the phagocytosis of cellular debris and activation of immune system [6, 46], extended activation may hinder neurogenesis and contribute to additional neuronal loss and unselective clearance of healthy tissue [8, 41]. In cluster 1 microglia trajectories, the M2 response appears to persist along with M1 activity but is not elevated enough to keep M1-induced secondary damage suppressed via compensatory pro-health mechanisms.

Of note, the mean age of cluster 1 individuals (46.09 ± 3.26 year) was significantly higher than both cluster 2A and 2B indicating that there may be age-related dysfunctions in microglia involved after TBI. With increasing age, microglia morphology changes and functional impairments are observed. Microglia are found in less ramified form with altered cytokine receptor patterns which may hinder their ability to respond appropriately to inflammatory stimuli [33, 40, 44]. In addition to an already elevated baseline inflammatory state in aged individuals, surveying microglia cells lean toward a “primed” phenotype characterized by activation at a lower threshold, tendency to adopt an exaggerated pro-inflammatory phenotype, and resistance to regulatory anti-inflammatory cues [6, 40]. In cerebral ischemia injury models, stress has similarly been shown to contribute to microglial priming which may exacerbate inflammatory dynamics following brain injury [47]. Stress-related implications likely arise in cluster 1, as acute CSF cortisol levels are significantly elevated compared to the other clusters. These findings are in line with previous work showing that exaggerated inflammatory responses, especially among aged individuals, are associated with elevated acute CSF cortisol levels and TBI mortality [19, 38].

Additionally, mean IL-10 levels in cluster 1 were significantly higher than both cluster 2A and 2B (57.54 vs. 8.08 and 11.54 pg/mL, respectively) over the first 5 days post-TBI. Despite increased anti-inflammatory presence, pro-inflammatory production levels and microglia activity persisted perhaps due to reduced sensitivity of microglia to anti-inflammatory mediation [40] and insensitivity of cytokine release to anti-inflammation (elevated *a*_*∞*2_ in our model). Our findings for cluster 1 are consistent with previous studies that found associations between CSF IL-10 levels, age, and mortality rates [48, 49].

#### Cluster 2B—evidence of transient inflammation

In contrast to cluster 1, both M1 and M2 microglia and IL-10 levels decrease early and rapidly in cluster 2B. Pro-inflammatory mediator levels remain elevated but plateau, providing little to no re-initiation of microglial activation and polarization once the levels fall to baseline. There is a late rise in IL-1β that appears over day 3, possibly due to secondary tissue damage release, but the model could not capture an elevation of such low magnitude. This rise in IL-1β does, however, fit nicely to the late IL-12 rise. The consistent pro-inflammatory expression through day 5, coupled with the rapid decreases of IL-4 and IL-10 and greater sensitivity to IL-10 inhibition of further cytokine, leads to the elevated tissue damage in cluster 2B model ensembles.

The most elevated levels of the tissue damage term are observed for cluster 2B. This qualitative evidence from our modeling efforts supports the concept that the complete suppression of the neuroinflammatory response and microglial activity, of either phenotype, is potentially detrimental to tissue recovery post-TBI [5, 6]. In the absence of adequate microglia activity over the first 5 days, damage from the initial injury may not be addressed, leading to further damage and perpetuating other secondary injury cascades.

By PCA and cluster analysis [18], cluster 2A and 2B were indistinguishable when considering day 0–3 neuroinflammatory profiles. However, when considering CSF hormone data, we found that cluster 2B individuals were characterized by significantly higher cortisol levels than cluster 2A over the first 3 days post-TBI, consistent with differences in cortisol trajectories group membership found between patient groups in previous work [38]. Elevated cortisol immediately following injury potentially contributes to the premature immunosuppression observed with cluster 2B, leading to a sub-physiological microglial response.

### Favorable outcome group (cluster 2A)—neuroprotective features of neuroinflammation

Cluster 2A model ensembles best demonstrate a beneficial physiological response to TBI. Microglial activity was present for a length of time that was neither permissive nor indiscriminate in pro-inflammatory mediated damage (as in cluster 1), or sub-physiological in minimizing tissue damage associated with other forms of secondary injury (as in cluster 2B). This observation is reflected in significant parameter differences regarding the decay of microglia and IL-10, as well as release rates of the pro-inflammatory cytokines. Although we observe a gradual increase in the damage expression, it is lower in magnitude than both unfavorable outcome clusters and appears to be well contained as it plateaus near day 5. Particularly of note, the relative ratio of M2:M1 microglia in cluster 2A was approximately 1:1 after the initial injury, becoming larger than 1 as the microglial response progressed. Conversely, M2:M1 ratios in clusters 1 and 2B were nearly 1:4 initially and became even smaller over time. These findings may support the physiological importance of microglia, even perhaps of the stereotypically neurotoxic M1-like phenotype, in the acute injury phase post-TBI.

### Challenges and limitations of modeling microglial physiology

There is considerable need in the TBI field to characterize neuroinflammation, particularly with respect to the contributions of microglial functionality, in order to assess acute injury progression and tailor intervention strategies to enhance neuroprotection for particular patient subgroups. In this study, we implemented CSF cytokine time courses as proxies to indicate the state of neuroinflammation post-TBI and inform microglia activation and polarization dynamics. However, the consistent collection of informative neuroinflammatory data is not always clinically feasible. The refinement of cerebral microdialysis (CMD) and CSF cytokine measurement methodology will contribute to ongoing efforts to centrally monitor TBI-induced neuroinflammation [50].

While cytokine data was available from a large patient cohort, more limited samples were available to contribute to each 6-h smoothed mean. Serial sampling and cytokine data at a greater temporal resolution would improve model trajectories to provide more accurate predictions of the progression of neuroinflammation post-TBI. The inflammatory data remained oscillatory in nature despite a smoothing procedure for averaging. Marked changes in inflammatory mediator dynamics were largely absent, with most levels present at consistently low levels. As a result, the fits to cytokine data did not produce noticeable fluctuations between microglia phenotypes.

There is inherent abstraction in mathematically modeling biological processes. In our model, microglia subtypes and tissue damage are qualitatively projected with arbitrary units rather than quantified by cell count or tissue volume. This limits the direct interpretation of these time courses to an estimate of the M1- or M2-like “state” of the brain following injury. Moreover, parameter values cannot be taken as literal rates with established units. Although model parameters were initially guided by existing experimental literature, scaling procedures on cytokine data and confounding factors in experimental situations complicate the direct rate interpretations. Our determination of differences between parameter value distributions should be viewed as a relative test of neuroinflammatory distinctions between clusters, which integrated additional statistical metrics to inform the degree to which the distributions differed. Due to limitations of the statistical methodology, we in fact de-emphasized the value of traditional hypothesis testing alone and considered two Bhattacharyya metrics to inform our comparative parameter analyses. Parameter value differences were ranked by lowest overlap (BC) and highest spread (BD) to highlight the most dissimilar parameter distributions between clusters.

Additionally, as a reduced model, the set of ODEs derived were limited to prototypical markers of microglia activation and acute inflammation to encompass basic regulatory components in the inflammatory network: initiation, propagation, phenotype switching, and inhibition. The model was designed to represent recent findings that IL-4 and IL-10 act as switch-like factors in microglia polarization. However, the self-regulating and immunosuppressive role of these anti-inflammatory mediators may be overemphasized in this model, based on the relatively rapid return of microglia to baseline levels predicted in all clusters. Inclusion of additional mediators may create a more comprehensive and nuanced illustration of the inflammatory network that drives the microglial response to TBI.

In general, the understanding of microglia classification and roles is still evolving in the neurotrauma field. At first, a spectrum of activation states was proposed, ranging from M1 to M2 extremes, with multi-functional subtypes of M2 in between [46, 51]. Not only are more nuanced views now being considered as alternatives to these rigid phenotype classifications, a layer of complexity has been added as simultaneous expression of M1 and M2 phenotypic markers on the same cell has now been observed in animal models [9]. This developing research area calls for further classification of molecular profiles and associated functional roles of microglia, particularly advancements that successfully translate in vitro findings to in vivo scenarios. Macrophage research and dynamics have informed our ODEs and have paved the way for much of our understanding of phenotype polarization and functionality; however, it is necessary to investigate these parallels in the brain with microglia in order to extend M1 and M2 characterizations explicitly. A key strength of our work is that the modeling framework that we utilize need not be interpreted in terms of a strict M1/M2 dichotomy. Rather, the framework allows for a flexible interpretation of the M1 and M2 variables as cell counts, states, or even associated microglia functions. The focus is on the relative contribution and effects of the pre-specified cytokines on the model behavior of those variables as well as the physiological processes of pro- and -inflammatory cytokine production, tissue damage, and healing that M1 and M2 represent in our model; as long as these elements are present and interrelated in the biological response to damage, our predictions related to these quantities are not dependent on any specific M1/M2 dichotomy.

More generally, our model encodes physiological interactions among biological quantities thought to contribute to inflammatory response dynamics as well as to tissue damage and healing in the acute phase post-TBI. This framework requires making assumptions about which physiological processes contribute and in what ways. We acknowledge that alternative sets of assumptions could lead to different conclusions, but we have attempted to tailor our modeling choices to reflect current understanding derived from previous experimental, clinical, and computational work, albeit with some simplifications to retain tractability. Nonetheless, any experimental or clinical work aimed at predicting outcome, such as suggesting early-warning signs for patient risk groups and targets for therapeutic interventions, would necessarily proceed based on some theoretical framework, typically reflecting the prevailing scientific viewpoint, which would exert a strong impact on the study performed. Among the advantages of the modeling approach used here in this report, we note that the underlying assumptions are clearly stated (see “Ordinary differential equation model development” section) and the parameter fitting process does not impose any additional biases; rather, this modeling approach evaluates all model parameters’ contributions to cluster differences, giving all an equal chance to emerge as significant.

Lastly, microglia responses in the acute phase may be a transient phenomenon overshadowed by subsequent chronic elevations [46, 52], which have been observed as late as 11 months to 17 years post-TBI in humans [40], and accompanying pathologies; nonetheless, these transients may contribute to long-term influences of acute neuroinflammation on patient outcome [18].

### Stratifying patient subgroups for improved prognosis and treatment

PCA and cluster analysis by R.G. Kumar et al. identified a cluster of individuals (cluster 1) with elevated inflammation with respect PC1 markers. However, the outcomes of cluster 2 individuals in this study were variable, despite negative PC1 scores for the majority [18]. This work indicates that elevations of PC1 markers (IL-5, IL-6, IL-8, IL-10, sVCAM, sICAM, and sFAS) were predictive of outcome for a subset of the patients in the study; however, other predictive measures were yet to be unveiled to distinguish patient prognosis in cluster 2. In the current study, we grouped cluster 2 patients by 6-month GOS score to investigate differences in their inflammatory and microglial dynamics post-TBI beyond classification of particular acute inflammatory markers. Despite having greater cortisol levels in the first 3 days following TBI, cluster 2B individuals were also projected to be in an immunosuppressed state due to early microglia decay and greater sensitivity to IL-10 in negative feedback mechanisms that control cytokine production.

Our findings on cluster 2B emphasize the important point that microglial responses underlying poor patient outcomes post-TBI are likely heterogeneous; in particular, poor outcomes in some patients might relate to a sub-physiological microglial response. This interpretation of results exemplifies the utility of mathematical modeling in exploring how early patient stratification based on inflammatory marker expression may lead to informed understanding of acute recovery trajectories and guide decision-making for specific immunomodulatory therapy types that may benefit from additional pre-clinical evaluation.

## Conclusion

This modeling approach makes both clinical and computational contributions to the growing conceptualization of microglia pathophysiology following severe TBI. We have integrated statistical and mechanistic modeling to investigate potential sources of acute pathologies that lead to particular outcomes. This novel approach in TBI demonstrates the feasibility of computationally extracting predictions about intervention targets in a way that is informed by mechanistic understanding of the underlying physiology. In future studies, this mathematical modeling framework can serve as a manipulable system, via adjustment of target parameters that are of mechanistic importance to the neuroinflammatory system, to simulate pharmacological intervention effects and improve our understanding of neuroinflammatory kinetics. Our model can also be augmented to incorporate additional biological features that connect with available data. Our results predict that the early stratification of distinct patient subgroups based on neuroinflammatory differences could support personalized therapies that modulate the microglial response, including the balance and timing of transitions of M1- and M2-like states, or the associated inflammation-related processes, following TBI. Informing such computational approaches with new evidence on phenotypic markers and specific roles of each microglia subtype, as well as validating model predictions with additional patient data, will promote the development of TBI interventions that harness the multifaceted nature of neuroinflammation and microglia in a way that mitigates secondary injury and improves patient outcome.

## Additional file

Additional file 1: **Tables** **S1-S3.** Shown are the most dissimilar parameter distributions (determined by Bhattacharyya metrics and model sensitivity analysis) in pairwise cluster comparisons. The ranges and averages of each parameter distribution by cluster are listed to supplement the box and whisker plot representations in Fig. 5 of the main manuscript. (PDF 45 kb)

## Abbreviations

AIS: Abbreviated Injury Scale
BC: Bhattacharyya coefficient
BD: Bhattacharyya distance
BMI: Body mass index
CNS: Central nervous system
CSF: Cerebral spinal fluid
*D*: Secondary CNS tissue damage
DAMPs: Damage-associated molecular patterns
GCS: Glasgow Coma Scale
GOS: Glasgow Outcome Scale
ICD: International Classification of Disease
ICU: Intensive care unit
IL: Interleukin
IL1: IL-1β
IL4: IL-4
IL10: IL-10
IL12: IL-12
IQR: Interquartile range
ISS: Injury severity scale score
*M*1: M1-like microglia
*M*2: M2-like microglia
*Mr*: Resting microglia
ODE: Ordinary differential equation
PC: Principal component
PCA: Principal component analysis
*S*: Model sensitivity
SE: Standard error of the mean
sFAS: Soluble Fas
sICAM-1: Soluble intracellular adhesion molecule-1
sVCAM-1: Soluble vascular adhesion molecule-1
TBI: Traumatic brain injury
Th: T-helper
Th2: Type 2 T-helper
TNF-α: Tumor necrosis factor alpha
