# Supplementary material for: A mathematical model of neuroinflammation in severe clinical traumatic brain injury
Source: J Neuroinflammation. 2018 Dec 18;15:345. doi: 10.1186/s12974-018-1384-1 (PMC6299616; doi:10.1186/s12974-018-1384-1)
Supplement: Supplementary file 1 — Tables S1-S3. Shown are the most dissimilar parameter distributions (determined by Bhattacharyya metrics and model sensitivity analysis) in pairwise cluster comparisons. The ranges and averages of each parameter distribution by cluster are listed to supplement the box and whisker plot representations in Fig. 5 of the main manuscript. (PDF 45 kb) [file 12974_2018_1384_MOESM1_ESM.pdf]

Supplementary Table 1. Cluster 1 vs. 2A parameter value comparisons.

	Cluster 1		Cluster 2A	
Parameter	*Range*	*Average*	*Range*	*Average*
k_{pn1}	0.07 – 29.90	15.51	0.05 – 0.93	0.25
k_{pn12}	8.10 – 49.88	32.31	0.05 – 1.25	0.42
μ_{M1}	0.05 – 0.46	0.14	2.41 – 22.92	6.88
μ_{n10}	0.30 – 0.72	0.50	0.82 – 1.70	1.29
$a_{\infty 2}$	25.0 – 38.51	26.42	1.31 – 16.21	8.78

Distributions of model parameters representing the release rates of IL-1 (k_{pn1}) and IL-12 (k_{pn12}), decay rates of M1 (μ_{M1}) and IL-10 (μ_{n10}), and a threshold-like term for the effectiveness of IL-10 inhibition on further cytokine release ($a_{\infty 2}$), differed significantly between Clusters 1 and 2A.

Supplementary Table 2. Cluster 1 vs. 2B parameter value comparisons.

	Cluster 1		Cluster 2B	
Parameter	*Range*	*Average*	*Range*	*Average*
k_{pn1}	0.07 – 29.90	15.51	0.11 – 2.86	0.91
k_{pn12}	8.10 – 49.88	32.31	0.59 – 8.06	1.83
μ_{M1}	0.05 – 0.46	0.14	4.73 – 37.32	10.77
μ_{M2}	2.87 – 11.89	5.43	7.33 – 32.05	17.70
μ_{n12}	0.06 –0.63	0.26	0.050 – 0.076	0.052
μ_{n10}	0.30 – 0.72	0.50	1.18 – 1.40	1.30
h_n	1.03 – 6.00	5.14	1.00 – 2.23	1.27

Distributions of model parameters representing the release rates of IL-1 (k_{pn1}) and IL-12 (k_{pn12}), decay rates of M1 (μ_{M1}), M2 (μ_{M2}), IL-12 (μ_{n12}) and IL-10 (μ_{n10}), and Hill coefficient (h_n), differed significantly between Clusters 1 and 2B.

Supplementary Table 3. Cluster 2A vs. 2B parameter value comparisons.

	Cluster 2A		Cluster 2B	
Parameter	*Range*	*Average*	*Range*	*Average*
k_{pn12}	0.05 – 1.25	0.42	0.59 – 8.06	1.83
μ_{M2}	1.23 – 6.39	3.45	7.33 – 32.05	17.70
$a_{\infty 2}$	1.31 – 16.21	8.78	25.00 – 44.30	27.97
v_n	0.20 – 1.21	0.85	0.87 – 25.85	7.17

Distributions of model parameters representing the release rate of IL-12 (k_{pn12}), decay rate of M2 (μ_{M2}), a threshold-like term for the effectiveness of IL-10 inhibition on further cytokine release ($a_{\infty 2}$), and half-activation constant (v_n) differed significantly between Clusters 2A and 2B.
